# Estimating the long term impact of kidney donation on life expectancy and end stage renal disease

**DOI:** 10.1186/2047-1440-2-2

**Published:** 2013-02-16

**Authors:** Bryce A Kiberd

**Affiliations:** 1 Department of Medicine, Dalhousie University, Halifax, 5082 Dickson Building, Queen Elizabeth II HSC VG site. University Ave, Nova Scotia, B3H 1V8, Canada

**Keywords:** Quality of life, Nephrectomy, Live donation, End stage renal disease, Life expectancy

## Abstract

**Background:**

Long term studies of live kidney donation do not show evidence of appreciable risks to the donor. However nephrectomy reduces total glomerular filtration rates (GFR) and is associated with increased rates of proteinuria and possibly hypertension. It is not clear to what extent these changes are associated with reduced life expectancy (LE) or increased risk of end stage renal disease (ESRD) since follow up is incomplete in most reports.

**Methods:**

In a computer simulation model based on a US population chronic kidney disease model, increased hazard rates for higher blood pressure, proteinuria and low GFR were applied to healthy individuals undergoing donor nephrectomy. Subsequent LE and cumulative risk of ESRD were calculated.

**Results:**

Kidney donation is projected to reduce LE by 0.83 years and increase the absolute cumulative risk of ESRD by 0.89% for a 40-year-old white male. White females were predicted to have slightly greater loss of life and less added ESRD risk. Conversely, Blacks have greater risks of ESRD after donation. Older donors with hypertension were predicted to lose less life years and lower cumulative ESRD risks than young donors. Despite these increased risks most donors will have better life expectancy and lower ESRD rates than the general population since they are a highly selected cohort.

**Conclusions:**

This study attempts to quantify increases in death and ESRD from donor nephrectomy assuming the risk factors of hypertension, low GFR and proteinuria have the same significance in this population as in the general population. Further study is required to better estimate the risks of donation and test whether these assumptions are valid.

## Background

Live kidney donation is responsible for a considerable proportion of transplanted kidneys throughout the world [1]. Although long considered a relatively safe practice there has been renewed interest in studying the effects of donor nephrectomy. This interest has been generated from the use of donors who are older and who have medical abnormalities such as hypertension [2] and the growing evidence that reduced glomerular filtration rate (GFR) and proteinuria are strong independent predictors of cardiovascular disease and mortality as well as end stage renal disease (ESRD) [3,4]. As a result some advocate increased study of the effects of nephrectomy and long term follow up of donors to ensure that those who develop hypertension, proteinuria or diabetes mellitus have prompt intervention [5]. Analysis of donor registries would better quantify the incremental risks associated with donation which would help inform future potential donors.

The objectives of the analysis are to:

1. Estimate the impact of increases in blood pressure, proteinuria and reduced GFR on long term life expectancy and ESRD rates in healthy white males.

2. Examine differences in risk for white females and those of black men.

3. Estimate the impact of younger versus older healthy donors and those with medical abnormalities (hypertension and glucose intolerance) on long term life expectancy and ESRD rates.

## Methods

A population model was created to examine the development of ESRD in the US population. The model was created in TreeAge and the entire tree [see Additional file 1: Figure S1] is available in the supporting information. The model incorporated health states of normal, hypertension, and diabetes mellitus. These states were modeled with and without proteinuria. In addition there were four health states of low GFR chronic kidney disease (CKD: CKD and diabetes mellitus with and without proteinuria and CKD and non-diabetes mellitus with and without proteinuria) and one state of ESRD. Different stages of low GFR CKD were not used but rather a composite state which included stage 3 and 4. Hazard risks for mortality and kidney disease progression were taken from the literature [see Additional file 1: Tables S1 and S2, [6-11]]. Mortality in the general and ESRD population were taken from published vital statistics and the US Renal Data System (USRDS), respectively [12,13]. The model assumed that all 20-year-old males were initially completely normal. Outputs were calibrated to population prevalences for hypertension, proteinuria (Stage 1 and 2 CKD), low GFR CKD, and diabetes mellitus [see Additional file 1: Table S3, [14-17]]. The US 2000 standard population was used to calculate population prevalence [18,19]. The model was also calibrated to reproduce the life expectancy of a 20-year-old white male (56.6 years) and the cumulative risk of ESRD (3.5%) as determined by USRDS annual incidence rates over 100 years of potential life [12,13]. The model closely matched observed overall population survival and ESRD rates [see Additional file 1: Figures S2 and S3]. Similar models were developed for white females and black men in the US [see Additional file 1: Tables S4 and S5]. Since cumulative risks of ESRD are similar for black men and women a separate model was not created.

GFR, proteinuria and blood pressure changes as a result of live donor nephrectomy were taken from a recent meta-analysis of the literature [20,21]. These studies report that systolic blood pressure increases by 5 mm Hg and that 12% of donors develop proteinuria within a short time period. The analysis assumes patients who have an increase in blood pressure or proteinuria will assume the same increased risks of those in the general population with higher blood pressure and proteinuria. Nephrectomy also reduces GFR and this increases the risk of eventually reaching a low GFR of <60 ml/min/1.73m^2^ over time [21]. Those who develop a reduced GFR <60 ml/min/1.73m^2^ will also be at an increased risk of death and ESRD. All three factors (lower GFR, higher blood pressure and proteinuria) were examined separately to assess the impact of nephrectomy on life expectancy and cumulative ESRD risk. Given the small and uncertain effects of the increase in blood pressure the combined impact of proteinuria and reduced GFR was examined. Since the mean age of a donor in the US is approximately 40 years, this was the reference age. A sensitivity analysis [see Additional file 1: Table S6] was also performed using higher hazard ratios for CKD progression and mortality associated risk factors (proteinuria and hypertension) on pre- and post-donation life expectancy and cumulative ESRD. The effect on quality of life was also examined using published quality of life utilities for disease states [see Additional file 1: Table S7].

## Results

Table 1 shows the individual and combined factor effects on life expectancy and cumulative ESRD. Reduced GFR has the largest impact on ESRD risk, whereas proteinuria has the largest effect on life expectancy. Increased blood pressure had the smallest effect on ESRD. These findings were consistent across age and ethnicity.

**Table 1:** Model prediction of an increase in blood pressure, proteinuria and reduction in GFR from nephrectomy on change in life expectancy and cumulative ESRD risk in 40-year-old donors

	**↑ 5 mm HG systolic BP**			**↑ 12% post donation proteinuria**			**Reduced GFR**			**Combined GFR and proteinuria effects**		
Change	WM	WF	Black	WM	WF	Black	WM	WF	Black	WM	WF	Black
Δ Life Expectancy years	−0.30	−0.31	−0.33	−0.63	−0.65	−0.60	−0.20	−0.22	−0.32	−0.83	−0.88	−0.91
Δ Cum ESRD %	+0.01	+0.01	+0.03	+0.32	+0.24	+0.56	+0.52	+0.34	+1.10	+0.89	+0.63	+1.67

BP, blood pressure; Cum, cumulative; GFR, glomerular filtration rate; WF, white females; WM, white males.

**Figure 1:**
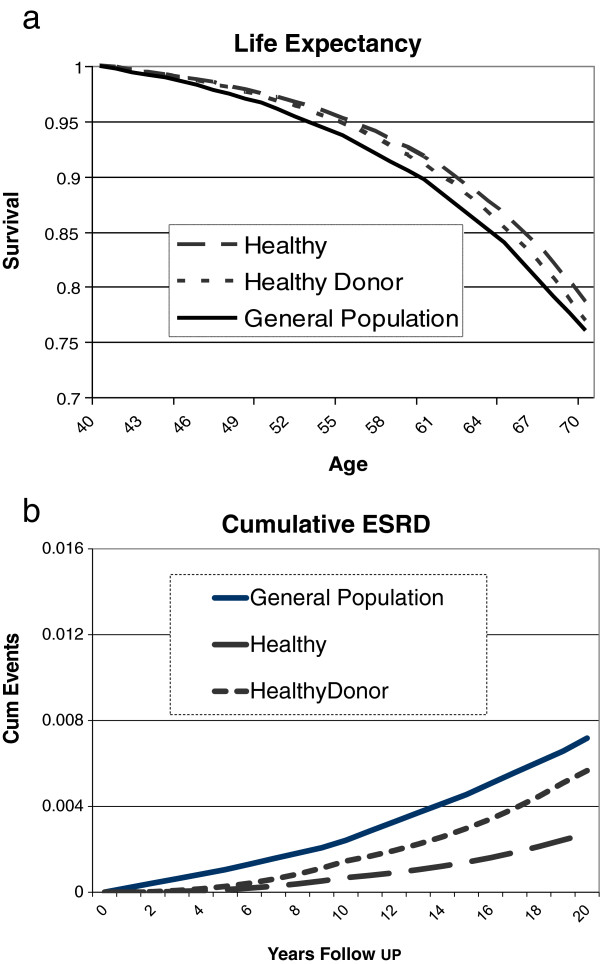
**a.Patient survival in 20-year-old white normal/general population male and the effect of donor nephrectomy.b.** Cumulative risks of ESRD for a 20-year-old white normal/general population male and the effect of donor nephrectomy.

Tables 2, 3, 4 show the combined (increase in proteinuria and reduced GFR) effects in healthy potential donors of various ages. In all groups nephrectomy is associated with lower absolute cumulative risks in older subjects compared to the youngest (20 years old). White women suffer slightly greater loss of life but lower incremental risks of ESRD compared to white men. Black men suffer the greatest cumulative risks; however, they also have the highest baseline risk. Figure 1a and 1b show patient survival and cumulative risk of ESRD for 40-year-old white men in the general population, healthy subjects and healthy subjects who have donated a kidney. Most of the increase in events occurs late post donation. As shown, patient survival is better and cumulative risks of ESRD are lower in donors compared to the general population but worse in healthy non-donors.

**Table 2:** Model prediction for the impact of nephrectomy on life expectancy and cumulative risk of ESRD in 20, 40, 50 and 60 year old white males

	**20 years**	**40 years**	**50 years**	**60 years**
Healthy normal without donation				
LE years	56.6	38.53	29.95	22.05
Cum ESRD %	3.51	2.60	1.96	1.44
Risk difference after donation				
Δ LE years	−0.92	−0.83	−0.77	−0.72
Δ Cum ESRD	+1.01	+0.89	+0.67	+0.50
Added Risk of ESRD	1/99	1/112	1/150	1/200

Cum, cumulative; ESRD, end stage renal disease; LE, life expectancy.

**Table 3:** Model prediction for the impact of nephrectomy on life expectancy and cumulative risk of ESRD in 20, 40, 50 and 60 year old white females

	**20 years**	**40 years**	**50 years**	**60 years**
Healthy normal without donation				
LE years	61.13	42.56	33.64	25.23
Cum ESRD %	2.33	1.62	1.14	0.78
Risk difference after donation				
Δ LE years	−0.94	−0.88	−0.83	−0.81
Δ Cum ESRD	+0.80	+0.63	+0.45	+0.31
Added ESRD Risk	1/125	1/159	1/222	1/326

Cum, cumulative; ESRD, end stage renal disease; LE, life expectancy.

**Table 4:** Model prediction for the impact of nephrectomy on life expectancy and cumulative risk of ESRD in 20, 40, 50 and 60 year old black males

	**20 years**	**40 years**	**50 years**	**60 years**
Healthy normal without donation				
LE years	51.13	33.98	26.46	20.13
Cum ESRD %	8.58	6.51	4.49	2.98
Risk difference after donation				
Δ LE years	−1.05	−0.91	−0.85	−0.82
Δ Cum ESRD	+1.75	+1.67	+1.37	+0.99
Added ESRD Risk	1/57	1/60	1/73	1/100

Cum, cumulative; ESRD, end stage renal disease; LE, life expectancy.

The study also examined 50-year-old male donors with hypertension and those at a two-fold increased risk of diabetes mellitus. Table 5 shows that both of these groups with medical abnormalities have shorter baseline life expectancies and higher cumulative risks of ESRD without donation. The absolute increase in risks from donation was slightly greater for these donors compared to healthy 50-year-old donors but was comparable to the incremental risks taken by a 20-year-old normal donor.

**Table 5:** Model prediction for the impact of nephrectomy on life expectancy and cumulative risk of ESRD in 50-year-old white males with and without medical abnormalities

	**50 year old white males**		
	Healthy	Hypertension	Increase risk of diabetes mellitus
Life expectancy years, Cum ESRD %	29.96, 1.96	28.6, 2.12	28.84, 2.93
	Risk difference after donation		
Δ Life expectancy years	−0.77	−0.85	−1.06
Δ ESRD %	+0.67	+0.69	+1.12
Added Risk of ESRD	1/150	1/145	1/90

ESRD, end stage renal disease.

## Discussion

This analysis shows that nephrectomy is predicted to reduce life expectancy and increase the risk of ESRD. Although the focus has been on the added risk of ESRD, the study also quantifies an increase in death associated with low GFR and proteinuria.

The study shows that women are predicted to incur slightly greater reductions in life expectancy with lower added risks of ESRD with donation compared to men. Black male donors not only are at increased absolute risk of ESRD but the incremental risk from donation is also greater. It is not surprising that a significant proportion of patients who were prior kidney donors and subsequently developed ESRD would be Black [22,23]. As postulated by Steiner, older donors are likely to have lower cumulative risks of ESRD compared to their younger counterparts [2,24]. According to a published survey there is center to center variation in what is an acceptable GFR [25]. Donors with lower baseline GFRs (GFR 60 to 80 ml/min pre-donation) could have even higher cumulative risks of ESRD.

The model also shows, as postulated by Steiner, that older patients with hypertension may be acceptable donors [2]. Although these donors are at increased risk of death and ESRD at baseline the incremental risks may be the same or lower than younger perfectly healthy donors.

There are significant limitations to the analysis. This analysis may well overestimate the risk associated with donation and extreme caution should be taken in the interpretation of this data. Predicting very late events is problematic and much of the loss of life and risk of ESRD occurs after many years of follow up at the time of greatest uncertainty. It would be premature to use these estimates in counseling potential donors at this time. A sensitivity analysis [see Additional file 1: Table S6] demonstrates small absolute differences in the estimates despite significant increases in selected hazard ratios for proteinuria and reduced GFR. Although the absolute estimates are in question, the trends, such as higher cumulative risks in the young compared to older persons and differences by sex and race, are likely true. Secular trends in diagnosis and treatment may well change parameter estimates. As blood pressure control in the population improves, the effect of hypertension on life expectancy may diminish further. At this point, the analysis shows the increase in systolic blood pressure of 5 mm Hg was predicted to have a relatively small impact (−0.33 years of life expectancy and +0.01% cumulative risk of ESRD). Therefore, adding the increase in blood pressure to the combined risks of proteinuria and reduced GFR into the model will have almost no effect on the cumulative risk of ESRD but will increase lost years by approximately 0.33 years. Most important is the assumption that proteinuria generated from nephrectomy carries the same significance as proteinuria generated from hypertension, diabetes mellitus, primary renal disease or genetics. One might expect that proteinuria as a result of a sudden reduction in renal mass would likely have the same prognostic significance on subsequent progressive kidney function loss compared to proteinuria generated from other causes. Mechanistically proteinuria itself is postulated to cause renal scarring [26]. On the other hand, one might argue that proteinuria generated primarily as a result of a sudden reduction in renal mass will not have the same cardiovascular impact compared to proteinuria that is caused by systemic processes, such as hypertension and diabetes mellitus. Although suitably sized studies will be able to quantify changes in GFR, proteinuria and blood pressure post donation, determining the impact on hard endpoints (death and ESRD) will require large numbers followed for very long time periods. It is possible that other endpoints such as cardiovascular events might be suitable. Proteinuria has a high day to day variation; even in the population estimates, the numbers assumed to have fixed low grade proteinuria are 50% of those detected from a single sample [14]. Studies following live kidney donors should have strict criteria for diagnosing proteinuria (repeat testing) as this was a sensitive estimate for later events. A recent study suggested that the new onset proteinuria may stabilize rather than continue to increase over time [27].

There have been several large recent follow up studies on donors. The most recent retrospectively examined 56,458 donors in the US followed for on average 9.8 years and detected 126 cases of ESRD for a crude rate of 134 cases per million years at risk [23]. Unfortunately, there were no healthy controls; however, the authors estimated the rate in the general population would have been 354 cases per million years. An earlier study examined patient survival in US donors compared to the NHANES III population as normal controls [28]. This study actually showed that donors had statistically better patient survival than their matched controls. It is not clear why nephrectomy would confer a survival advantage. Although donors were matched, it is likely that other confounding variables were missed. For example, it is not clear that subjects in the control population were excluded if they had proteinuria. A third large study examined a more selected cohort of donors who received medical coverage from a US health maintenance organization [29]. This study showed that donors were at risk of developing CKD, diabetes and hypertension at least as frequently as a control population adjusting for important covariates. In a recent Canadian study of 2,028 live donors followed for a median of 6.5 years, a composite endpoint of death and cardiovascular events was lower in donors (hazard ratio (HR 0.66, 95% confidence interval (CI), 0.48 to 0.90) than in 20,280 matched [1] healthy controls [30]. As above, it is not clear why donors would be at a lower risk. Controls were not formally evaluated to donate but rather were selected based on a lack of abnormal reports from administrative databases. This control sample may not be equivalent to a truly acceptable kidney donor patient. This study is likely underpowered to detect differences in death as the survival curves in healthy donors and non-donors are virtually superimposed over the first 10 years. All the studies above and this analysis agree that there appears to be no increase in risk in the average donor compared to the general population. Without rigorous controls, very large sample sizes and meticulous follow up, these observational studies will miss modest incremental risks that may not become apparent for many years.

It is not clear whether this analysis will generate more or less interest in registries that follow donors closely with an eye to early intervention. Some have argued that there is a moral obligation to follow donors even if the event rates are low [3]. Whereas some feel that since the risks are low, the costs of this added follow up would not be a wise use of limited health care resources. Most would agree that potential donors should be counseled that they are at some increased risk from donation and that follow up is important. Treating donors with isolated hypertension (no proteinuria or low GFR) should follow recommended guidelines, but whether the target should be at the general population or CKD target is unclear [31]. Screening for proteinuria in the general population has been controversial. The PREVEND study has shown that angiotensin converting enzyme inhibition (ACEi) therapy in albuminuric patients reduces cardiovascular events [32]. However, some of these participants had hypertension, diabetes mellitus and prior cardiac events and the results did not reach significance except in a subgroup with a greater degree of proteinuria. In addition, event rates are likely to be lower in younger highly screened donors and treating normotensive low level proteinuric patients with ACEi may require large numbers to treat to prevent an event. In a recent systematic review by the US Preventive Services Task Force screening and treating patients with proteinuria in the absence of hypertension, cardiovascular disease or diabetes mellitus was uncertain [33].

Since overall ESRD incidence rates are considerably higher (almost two-fold) in the US population even when adjusted for ethnicity, these estimates are not relevant to many other countries [13]. The baseline probabilities rely on the validity of databases that are far from perfect. The outcomes presented are means, such that for many, donation will have no adverse impact. However, for some, the impact may be late and of little consequence or early and potentially of great consequence. Most medical decision analyses examine discounted life years. However, discounting cumulative risks of ESRD does not make sense. One alternative is to quantify the time spent with ESRD for the average person. The model predicts that the average 40-year-old male will spend his last 24.2 days with ESRD. Those who donate spend an additional 17.6 days with ESRD (total 41.8 days). Using a 3% discount rate, the number of ESRD days for the 40-year-old non-donor and donor are 9.8 and 17.5 days, respectively. Many medical decision analyses also examine discounted Quality of Life adjusted years {QALYs). The same 40-year-old healthy non-donor is expected to have 21.514 QALYs (38.53 undiscounted life years). Should he donate, the model predicts his QALYs fall to 21.188 (a difference of 0.326 or 1.5% of the total). Some have argued that the act of donation is a positive event. However, this potential benefit is not included in the model. One recent study found no significant effect on quality of life with donation [34]. It is not clear in a power analysis of that study what clinically relevant difference could be detected. In addition, that study did not examine the consequences if this person was not allowed to donate and their prospective recipient had no other live options. The extent that the act of donation increases an individual’s quality of life or prevents a decline in quality of life should be considered in the decision process. Another study from this group showed that many donors appear willing to take significant risks [35]. Since the upfront mortality from donation is very small (<5/10,000) and the reduced quality of life in the early post nephrectomy period transient, these were not added into the model.

## Conclusions

In summary, this analysis gives some estimate of the potential long term risks of kidney donation. The study shows that the ESRD risks are less than non-donors in the general population. It identifies the challenges to quantify this risk more precisely. It also provides some rationale for accepting older donors with mild hypertension. Further research is needed to determine the significance of proteinuria on long term outcomes in kidney donors. A substantial long term effort is required to determine accurately to what extent nephrectomy causes an increase in cardiovascular disease, all cause mortality and ESRD.

## Abbreviations

ACEi: Angiotensin converting enzyme inhibition; CKD: Chronic kidney disease; Cum: Cumulative; ESRD: End stage renal disease; GFR: Glomerular filtration rate; LE: Life expectancy; QALY: Quality adjusted life year.

## Competing interests

The author declares he has no competing interests.

## Supplementary Material

Additional file 1 **Figure S1.**Markov Model Tree. **Figure S2.** General Population and Modeled (Normal) Survival from Age 20 in White Males. **Figure S3.** General Population and Modeled (Normal) Cumulative ERSD from Age 20 in White Males. **Table S1.** Mortality Hazards for Disease States. **Table S2.** Progression to ESRD for Disease States. **Table S3.** Model Calibration 20 y/o White Male. **Table S4.** Model Calibration 20 y/o White Female. **Table S5.** Model Calibration 20 y/o Black Male. **Table S6.** Quality of Life Adjustments. **Table S7.** Sensitivity Analysis (Worse case Table S1 and S2). Click here for file
