# Supplementary material for: Estimating the long term impact of kidney donation on life expectancy and end stage renal disease
Source: Transplant Res. 2013 Feb 16;2:2. doi: 10.1186/2047-1440-2-2 (PMC3577426; doi:10.1186/2047-1440-2-2)
Supplement: Additional file 1 — Figure S1.Markov Model Tree. Figure S2. General Population and Modeled (Normal) Survival from Age 20 in White Males. Figure S3. General Population and Modeled (Normal) Cumulative ERSD from Age 20 in White Males. Table S1. Mortality Hazards for Disease States. Table S2. Progression to ESRD for Disease States. Table S3. Model Calibration 20 y/o White Male. Table S4. Model Calibration 20 y/o White Female. Table S5. Model Calibration 20 y/o Black Male. Table S6. Quality of Life Adjustments. Table S7. Sensitivity Analysis (Worse case Table S1 and S2). [file 2047-1440-2-2-S1.doc]

Supporting Information

Index

Figure S1. Markov Model Tree

Figure S2. General Population and Modeled (Normal) Survival from Age 20 in White Males.

Figure S3. General Population and Modeled (Normal) Cumulative ERSD from Age 20 in White Males

Table S1. Mortality Hazards for Disease States

Table S2. Progression to ESRD for Disease States

Table S3. Model Calibration 20 y/o White Male

Table S4. Model Calibration 20 y/o White Female

Table S5. Model Calibration 20 y/o Black Male

Table S6. Quality of Life Adjustments

Table S7. Sensitivity Analysis (Worse case Table S1 and S2)

References for Supporting Information

Figure S1. Markov Model Tree


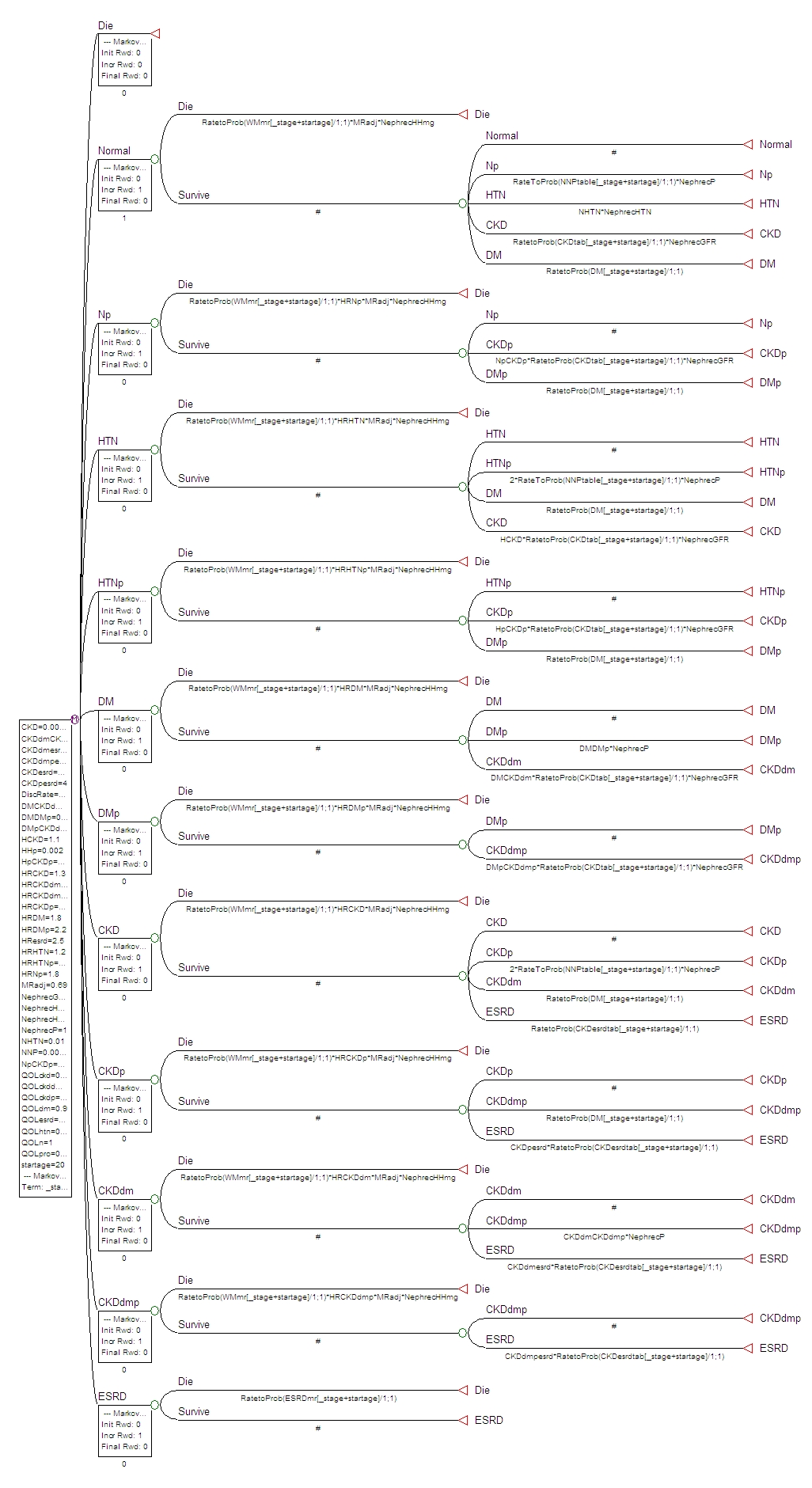


Figure S2. General Population and Modeled (Normal) Survival from Age 20 in White Males. Normal (Modeled) versus Observed (General Population)

Figure S3. General Population and Modeled (Normal) Cumulative ESRD from Age 20 in White Males using incidence rates from USRDS (1) to compare Observed (USRSD) versus Model (Normal).

Calculated from annual ESRD incidence rates from reference 1.

Table S1. Mortality Hazards for Disease States

| State | Mortality  (worse case) | Reference |
| --- | --- | --- |
| Normal | Reference State  HR 1.0 |  |
| Hypertension  Per 10 mmHg systolic | 1.2  1.05 | Weiner AJKD 2006 (2) |
| Diabetes Mellitus alone | 1.8 | Weiner AJKD 2006 (2)  Palmer NDT 2008 (3) |
| Proteinuria (CKD 1-2) | 1.8 (2.0) | CKD Lancet 2010 (4) |
| Low GFR (CKD stage 3+) | 1.3 (1.7) | Weiner JASN 2004 (5)  Hemmelgarn JAMA 2010 (6)  Go NEJM 2004 (7) |
| Combinations |  | |
| DM/Proteinuria | 2.2 | Palmer NDT 2008 (3)  CKD Lancet 2010 (4) |
| DM/CKD | 2.8 |
| DM/CKD/Proteinuria | 3.3 |
| CKD/Proteinuria | 2.8 |

Hazard ratios were preferentially taken from community based reports (Weiner) rather than CKD studies. Alternative hazard ratios for CKD low GFR and proteinuria were also examined using weighted (population size and presence of DM) estimates. (6-8)

Table S2. Progression to ESRD for Disease States

| State | Progression  N→CKD  (worse case) | Progression  CKD→ESRD | Reference |
| --- | --- | --- | --- |
| No DM/HTN/Proteinuria | Reference  HR 1.0 | Reference  HR 1.0 | Boulware JAMA 2003 (9)  Hoerger AJKD 2010 (10) |
| Proteinuria | 1.1 (2.0) | 4 |
| Hypertension (y/n)  Systolic 10 mm Hg | 1.1  1.05 | 1.4  1.05 |
| Diabetes Mellitus | 1.1 (2.0) | 2.8 |
| Hypertension/Proteinuria | 1.2 (2.2) | 4 |
| DM/Proteinuria | 1.1 (2.0) | 5.2 |

Table S3. Model Calibration 20 y/o White Male

|  | Observed | Model |
| --- | --- | --- |
| Life Expectancy | 56.6 yrs^11^ | 56.6 yrs |
| Hypertension  Prevalence | 0.29-0.305^12^ | 0.297 |
| DM diagnosed | 0.060-0.068^13, 14^ | 0.06375 |
| CKD Stage 1&2 | 0.044-0.051 ^15,16^ | 0.0491 |
| CKD Stage 3+ | 0.056-0.081 ^15,16^ | 0.0745 |

Adjusted to 2000 population (17, 18)

Table S4. Model Calibration 20 y/o White Female

|  | Observed | Model |
| --- | --- | --- |
| Life Expectancy | 61.1 yrs^11^ | 61.1 yrs |
| Cumulative ESRD | 0.0236^1^ | 0.0233 |
| Hypertension  Prevalence | 0.289 ^12^ | 0.304 |
| DM diagnosed | 0.045-0.054 ^13,14^ | 0.056 |
| CKD Stage 1&2 | 0.05-0.055 ^15,16^ | 0.0518 |
| CKD Stage 3+ | 0.07-0.095 ^15,16^ | 0.09 |

Total numbers in CKD Stage 1-4 is 0.121 to 0.150. Difficult to estimate individual CKD stage prevalence accurately from average data as presented in references.(6, 7, 15, 16)

Table S5. Model Calibration 20 y/o Black Male

|  | Observed | Model |
| --- | --- | --- |
| Life Expectancy | 51.3 yrs^11^ | 51.1 yrs |
| Cumulative ESRD | 0.0868^1^ | 0.0858 |
| Hypertension  Prevalence | 0.391 ^12^ | 0.393 |
| DM diagnosed | 0.090-0.98 ^13,14^ | 0.0982 |
| CKD Stage 1&2 | 0.04-0.055 ^15,16^ | 0.0454 |
| CKD Stage 3+ | 0.04-0.06 ^15,16^ | 0.0495 |

Total ^6, 7, 15, 16^ Stage 1-4 is 0.102 to 0.117. Difficult to estimate CKD stage prevalence accurately from average data as presented in references.

Table S6. Sensitivity Analysis (Worse case Table S1 and S2) 40 yo White Male

|  | Baseline | Worse Case Model* | Change |
| --- | --- | --- | --- |
| Life Expectancy | 38.53 yrs | 38.03 yrs | 0.050 yrs |
| Cum ESRD | 2.60% | 2.72% | 0.12% |
| Δ LE post donation | -0.83 yrs | -0.92 yrs | 0.09 yrs |
| Δ Cumulative ESRD post donation | +0.89 % | +0.92 % | 0.03% |

* Higher risks taken from Tables 1 and 2.

Table S7. Quality of Life Adjustments*

| State | Utility |
| --- | --- |
| No Diabetes mellitus/Hypertension/Proteinuria | 1.0 |
| Proteinuria | 1.0 |
| Hypertension | 0.98 |
| Diabetes Mellitus | 0.9 |
| CKD | 0.95 |
| CKD/diabetes mellitus/proteinuria | 0.85 |
| ESRD | 0.8 |
| Death | 0 |

* Adapted from Boulware JAMA 2003 (9) and Hoerger AJKD 2010 (10). Isolated proteinuria was not modeled to impact on utility scores.

References

1. U S Renal Data System, USRDS 2010 Annual Data Report: Atlas of Chronic Kidney Disease and End-Stage Renal Disease in the United States, National Institutes of Health, National Institute of Diabetes and Digestive and Kidney Diseases, Bethesda, MD, 2010.
2. Weiner DE, Tabatabai S, Tighiouart H, Elsayed E, Bansal N, Griffith J, Salem DN, Levey AS, Sarnak MJ: [**Cardiovascular outcomes and all-cause mortality: exploring the interaction between CKD and cardiovascular disease.**](http://www.ncbi.nlm.nih.gov/pubmed/16931212) *Am J Kidney Dis* 2006, **48**:392-401.
3. Palmer AJ, Valentine WJ, Chen R, Mehin N, Gabriel S, Bregman B, Rodby RA: [**A health economic analysis of screening and optimal treatment of nephropathy in patients with type 2 diabetes and hypertension in the USA.**](http://www.ncbi.nlm.nih.gov/pubmed/18359872) *Nephrol Dial Transplant* 2008, **23**:1216-23
4. Chronic Kidney Disease Prognosis Consortium, Matsushita K, van der Velde M, Astor BC, Woodward M, Levey AS, de Jong PE, Coresh J, Gansevoort RT: [**Association of estimated glomerular filtration rate and albuminuria with all-cause and cardiovascular mortality in general population cohorts: a collaborative meta-analysis.**](http://www.ncbi.nlm.nih.gov/pubmed/20483451) *Lancet* 2010, **375**:2073-81.
5. Weiner DE, Tighiouart H, Amin MG, Stark PC, MacLeod B, Griffith JL, Salem DN, Levey AS, Sarnak MJ: [**Chronic kidney disease as a risk factor for cardiovascular disease and all-cause mortality: a pooled analysis of community-based studies.**](http://www.ncbi.nlm.nih.gov/pubmed/15100371) *J Am Soc Nephrol* 2004, **15**:1307-15.
6. Hemmelgarn BR, Manns BJ, Lloyd A, James MT, Klarenbach S, Quinn RR, Wiebe N, Tonelli M; Alberta Kidney Disease Network: [**Relation between kidney function, proteinuria, and adverse outcomes.**](http://www.ncbi.nlm.nih.gov/pubmed/20124537) *JAMA* 2010, **303**:423-9
7. Go AS, Chertow GM, Fan D, McCulloch CE, Hsu CY: [**Chronic kidney disease and the risks of death, cardiovascular events, and hospitalization.**](http://www.ncbi.nlm.nih.gov/pubmed/15385656) *N Engl J Med* 2004, **351**:1296-305.
8. Levey AS, de Jong PE, Coresh J, El Nahas M, Astor BC, Matsushita K, Gansevoort RT, Kasiske BL, Eckardt KU: [**The definition, classification, and prognosis of chronic kidney disease: a KDIGO Controversies Conference report.**](http://www.ncbi.nlm.nih.gov/pubmed/21150873) *Kidney Int*  2011, **80**:17-28.
9. Boulware LE, Jaar BG, Tarver-Carr ME, Brancati FL, Powe NR. [**Screening for proteinuria in US adults: a cost-effectiveness analysis**.](http://www.ncbi.nlm.nih.gov/pubmed/14679273) *JAMA* 2003, **290**:3101-14
10. Hoerger TJ, Wittenborn JS, Segel JE, Burrows NR, Imai K, Eggers P, Pavkov ME, Jordan R, Hailpern SM, Schoolwerth AC, Williams DE; Centers for Disease Control and Prevention CKD Initiative: [**A health policy model of CKD: 1. Model construction, assumptions, and validation of health consequences.**](http://www.ncbi.nlm.nih.gov/pubmed/20116911) *Am J Kidney Dis* 2010, **55**:452-62.
11. Arias E: United States life tables, 2006. National vital statistics reports; vol 58 no 21. Hyattsville, MD: National Center for Health Statistics. 2010.
12. Ong KL, Cheung BM, Man YB, Lau CP, Lam KS: [**Prevalence, awareness, treatment, and control of hypertension among United States adults 1999-2004.**](http://www.ncbi.nlm.nih.gov/pubmed/17159087) *Hypertension* 2007, **49**:69-75.
13. Cowie CC, Rust KF, Byrd-Holt DD, Eberhardt MS, Flegal KM, Engelgau MM, Saydah SH, Williams DE, Geiss LS, Gregg EW: [**Prevalence of diabetes and impaired fasting glucose in adults in the U.S. population: National Health And Nutrition Examination Survey 1999-2002.**](http://www.ncbi.nlm.nih.gov/pubmed/16732006) *Diabetes Care* 2006, **29**:1263-8.
14. [www.cdc.gov/diabetes/statistics/prev/national/figraceethsex.htm](http://www.cdc.gov/diabetes/statistics/prev/national/figraceethsex.htm) accessed June, 23, 2011
15. [Coresh J](http://www.ncbi.nlm.nih.gov/pubmed?term="Coresh J"%5BAuthor%5D), [Selvin E](http://www.ncbi.nlm.nih.gov/pubmed?term="Selvin E"%5BAuthor%5D), [Stevens LA](http://www.ncbi.nlm.nih.gov/pubmed?term="Stevens LA"%5BAuthor%5D), [Manzi J](http://www.ncbi.nlm.nih.gov/pubmed?term="Manzi J"%5BAuthor%5D), [Kusek JW](http://www.ncbi.nlm.nih.gov/pubmed?term="Kusek JW"%5BAuthor%5D), [Eggers P](http://www.ncbi.nlm.nih.gov/pubmed?term="Eggers P"%5BAuthor%5D), [Van Lente F](http://www.ncbi.nlm.nih.gov/pubmed?term="Van Lente F"%5BAuthor%5D), [Levey AS](http://www.ncbi.nlm.nih.gov/pubmed?term="Levey AS"%5BAuthor%5D): **Prevalence of chronic kidney disease in the United States.** *[JAMA](http://www.ncbi.nlm.nih.gov/pubmed?term=coresh selvin  JAMA" \l "%23)* 2007, **298**:2038-47.
16. Coresh J, Byrd-Holt D, Astor BC, Briggs JP, Eggers PW, Lacher DA, Hostetter TH: [**Chronic kidney disease awareness, prevalence, and trends among U.S. adults, 1999 to 2000.**](http://www.ncbi.nlm.nih.gov/pubmed/15563563) *J Am Soc Nephrol* 2005, **16**:180-8.
17. Klein RJ, Schoenborn CA: *Age Adjustment Using the 2000 Projected U.S. Population*. Healthy People 2010 statistical notes: no. 20. Hyattsville, Maryland: National Center for Health Statistics. 2001.
18. [www.cbtrus.org/2010-NPCR-SEER/Tables2and3.pdf](http://www.cbtrus.org/2010-NPCR-SEER/Tables2and3.pdf) accessed June, 23, 2011
